# Open-Circuit Fault Detection and Classification of Modular Multilevel Converters in High Voltage Direct Current Systems (MMC-HVDC) with Long Short-Term Memory (LSTM) Method

**DOI:** 10.3390/s21124159

**Published:** 2021-06-17

**Authors:** Qinghua Wang, Yuexiao Yu, Hosameldin O. A. Ahmed, Mohamed Darwish, Asoke K. Nandi

**Affiliations:** 1 School of Mechatronic Engineering, Xi’an Technological University, Xi’an 710021, China; wqhhuazi@163.com; 2 Department of Electronic and Electrical Engineering, College of Engineering, Design and Physical Sciences, Brunel University, Uxbridge UB8 3PH, UK; yuyuexiao333@hotmail.com (Y.Y.); hosameldin.ahmed2@brunel.ac.uk (H.O.A.A.); mohamed.darwish@brunel.ac.uk (M.D.); 3 State Grid Sichuan Electric Power Research Institute of China, Chengdu 610094, China

**Keywords:** MMC-HVDC, fault detection, fault classification, LSTM, BiLSTM, CNN, classification accuracy

## Abstract

Fault detection and classification are two of the challenging tasks in Modular Multilevel Converters in High Voltage Direct Current (MMC-HVDC) systems. To directly classify the raw sensor data without certain feature extraction and classifier design, a long short-term memory (LSTM) neural network is proposed and used for seven states of the MMC-HVDC transmission power system simulated by Power Systems Computer Aided Design/Electromagnetic Transients including DC (PSCAD/EMTDC). It is observed that the LSTM method can detect faults with 100% accuracy and classify different faults as well as provide promising fault classification performance. Compared with a bidirectional LSTM (BiLSTM), the LSTM can get similar classification accuracy, requiring less training time and testing time. Compared with Convolutional Neural Networks (CNN) and AutoEncoder-based deep neural networks (AE-based DNN), the LSTM method can get better classification accuracy around the middle of the testing data proportion, but it needs more training time.

## 1. Introduction

Modular multilevel converters (MMCs) have been widely applied due to their advantages of modularity, extensibility, high-quality output, and high efficiency [1,2,3]. An MMC is formed by cascading multiple sub-modules (SMs) with the same structure. In a high voltage direct current (HVDC) transmission power system, the numbers of SMs are always up to several hundreds or thousands, which may induce some faults of SMs more likely to arise under complex and harsh conditions. The most application of SM circuits is the half-bridge circuit topology (HB-SM), which consists of two wire-bound insulated gate bipolar transistor (IGBT) modules along with their corresponding antiparallel diodes and a capacitor [4,5]. The HB-SM is commonly used due to its simplicity in terms of component count, lower losses, and ease of control. However, the main disadvantage in HB-SM is that it cannot provide blocking against DC fault. IGBT damage is the most common cause of sub-module failure [6], generally due to short-circuit faults or open-circuit faults [7]. Compared to the IGBT short-circuit fault, the IGBT open-circuit faults can last for a long time without being detected, which can deteriorate the output of the MMCs and can make the capacitors in the faulty SMs over-charged [8]. Therefore, this paper is concerned with the IGBT open-circuit fault diagnosis of Modular Multilevel Converters in High Voltage Direct Current (MMC-HVDC) systems.

Recently, several fault diagnosis methods have been discussed for the MMCs. These methods can be categorized into hardware-based and software-based methods [5]. The hardware-based methods are not suitable for the MMCs in HVDC systems because of the large number of the SMs in the MMC. Software-based methods can further be categorized into model-based methods and signal processing-based methods [9,10], according to whether the monitoring characteristics are inner characteristics or output characteristics [11]. The observers such as Luenberger observer [12], sliding mode observer [13,14], and Kalman filter observer [15,16], are prevalent model-based methods used to provide the detection references. Signal processing-based methods have been considered reliable and effective by several researchers [17,18,19,20] in recent years. However, both model-based and signal processing-based methods need to obtain suitable and appropriate inner features or thresholds of specific derived indices, such as zero-crossing current slope or harmonic content, which can degrade the robustness of fault diagnosis.

An alternative to traditional software-based approaches, artificial intelligence-based methods have been developed, which provide powerful tools to extract useful information for fault diagnosis based on historical data. Neural networks (NNs), one of the most basic artificial intelligence methods, have been used to detect a fault condition in the HVDC systems [21,22,23]. However, NNs need lots of training data and training time. Support vector machine (SVM) [24] and its optimization algorithms [25,26,27,28] have been employed to diagnose the faults of MMC. The support tensor machine (STM) [29], a generalization of the SVM, has been introduced to detect faults for MMC. However, in real-world applications, these artificial intelligent methods depend on feature extraction techniques. The quality of feature extraction directly affects the accuracy and efficiency of fault diagnosis.

Deep learning methods can avoid the problems of feature extraction, but the related publications are very limited in the application of MMC-HVDC systems. Convolutional Neural Networks (CNN) [30,31], and 1-D CNN [32] are proposed for fault classification and fault location in MMC-HVDC. Our research group proposed CNN, AutoEncoder-based deep neural network (AE-based DNN), and SoftMax classifier for MMC [33], the results showed that these deep learning methods have good potential. It is worth noting that the fault diagnosis of MMC so far has been mostly concerned on model-based research [34,35,36,37], less on data-driven diagnosis methods [38], and only some pioneering work has arisen in the publications about deep learning fault diagnosis of MMC.

Therefore, to develop a new deep learning method used for IGBT open-circuit fault diagnosis of MMC-HVDC systems to shorten such a gap, we aim to provide an LSTM approach to address the above-mentioned problems. The main contributions of this paper are outlined below.

(1) The proposed method has the ability to achieve accurate detection and classification of IGBT open-circuit faults but also can reduce the computational cost of sensing and learning from a large number of measurements.
(2) Without data preprocessing or post-operations, the fault detection accuracy of 100% and excellent classification accuracy are achieved.
(3) Performance comparisons of LSTM, bidirectional LSTM (BiLSTM), CNN, and AE-based DNN in terms of fault detection, classification accuracy, and time spent on training and testing for IGBT Open-circuit fault diagnosis of MMC-HVDC are provided.

This paper is organized as follows: Section 2 describes the MMC open-circuit faults and simulation experiments. Section 3 introduces a Recurrent neural network (RNN) and LSTM. Fault diagnosis of MMC-HVDC systems with LSTM is evaluated in Section 4. Section 5 compares LSTM with BiLSTM, CNN, and AE-DNN methods. Conclusions are drawn in Section 6.

## 2. Preliminaries on MMC Open-Circuit Faults and Simulation Experiments

### 2.1. MMC Sub-Module and Open-Circuit Faults

**Figure 1:**
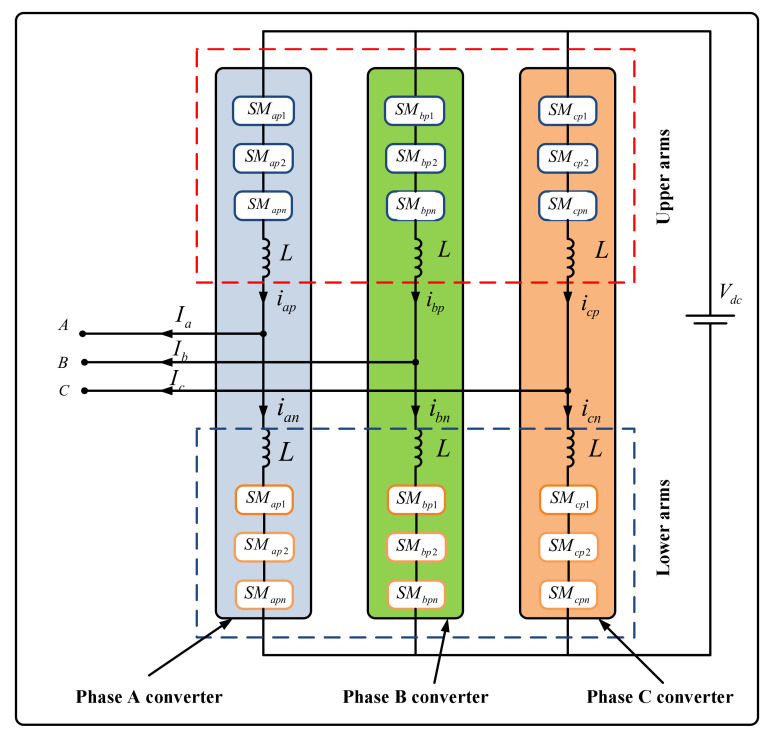
Structure of a three-phase MMC with half-bridge submodules [33].

**Figure 2:**
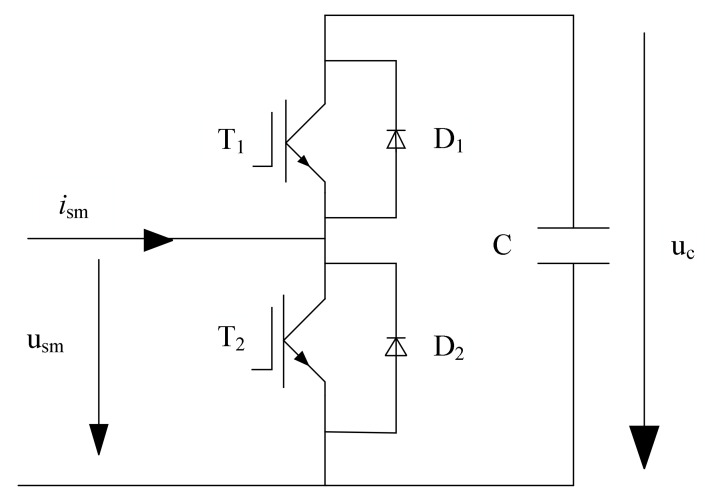
Circuit model of MMC sub-module.

A typical structure of a three-phase MMC consists of six arms as shown in Figure 1 [33]. Each arm consists of one inductor (L) and several identical SMs. Each SM involves one DC storage capacitor (C) and a half-bridge, which is composed of two IGBTs (i.e., $T_{1}$ and $T_{2}$) and two diodes ($D_{1}$ and $D_{2}$). The circuit of SM is shown in Figure 2.

Open-circuit faults of an SM can be sorted into $T_{1}$ fault and $T_{2}$ fault. When any fault occurs, the SM can be in ON ($s_{i}=1$) state or OFF ($s_{i}=0$) state, where $s_{i}$ is the corresponding switch function. Table 1 illustrates the output voltages of SM in different states for both normal and abnormal cases. In Table 1, $i_{sm}$ is SM current, $u_{c}$ is the capacitor voltage, and $u_{sm}$ is the output voltage of SM.

### 2.2. Simulation Experiments

In the PSCAD/EMTDC software environment, a two-terminal model of the MMC-HVDC transmission power system was simulated for this study. The system parameters of the operating environment and the MMC are shown in Table 2 [33].

The data recorded for this study are AC-side three-phase current ($I_{a},I_{b},I_{c}$) and three-phase circulation current ($I_{diffa},I_{diffb},I_{diffc}$). The circulation current and bridge current can be represented mathematically using the following equation:

(1) $$i_{diffk}=\frac{1}{2}\left(i_{kp}+i_{kn}\right)$$

where k stands for the a, b, and c phase, while p and n separately denote for upper and lower arms of the MMC. The symbols $i_{kp}$ and $i_{kn}$ are, respectively, the currents of the upper bridge and lower bridge of each three phases. Since the values of $i_{ap}$, $i_{bp}$, $i_{cp}$,$\;i_{an}$, $i_{bn}$, and $i_{cn}$ can be directly measured, we recorded them instead of $i_{diffa}$, $i_{diffb}$, and $i_{diffc}$. Consequently, we recorded nine parameters, i.e., $i_{a}$, $i_{b}$, $i_{c}$, $i_{ap}$, $i_{bp}$, $i_{cp}$,$\;i_{an}$, $i_{bn}$, and $i_{cn}$, (see Figure 1).

In our test, Table 3 [33] shows seven different health conditions of the MMC. In the processing of the seven states of the wind farm side MMC, the values of the nine parameters described above have been recorded. There are six types of faults occurring at different IGBTs at different times. These six types of faults were A-phase lower SMs, A-phase upper SMs, B-phase lower SMs, B-phase upper SMs, C-phase lower SMs, and C-phase upper SMs, at six different locations of IGBT break-circuit fault manually for each bridge. The total time of recording was 0.1 s while the time for the IGBT open circuit fault duration has been varied from 0.03 s to 0.07 s. The time step is 2 μs and the sampling frequency is 0.5 MHz. We collected 700 cases of seven different health conditions.

## 3. RNN and LSTM

**Figure 3:**
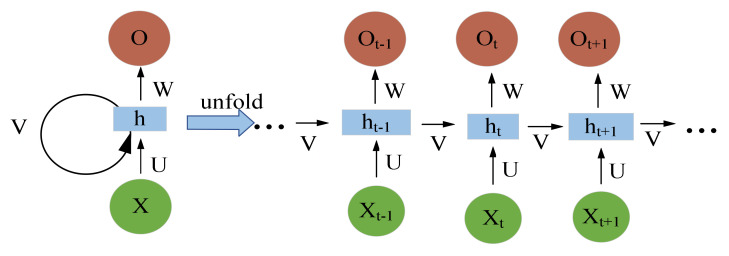
RNN structure.

**Figure 4:**
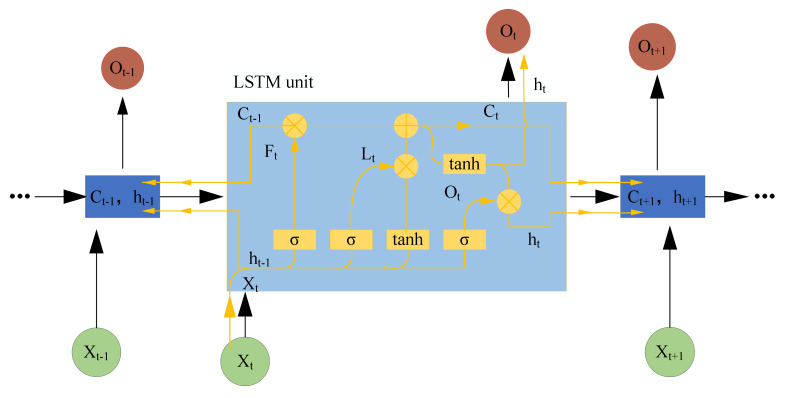
LSTM structure.

Recurrent neural network (RNN) has become one of the important subfields of deep learning, which has been widely used in the fields of speech recognition [39], rotating machine fault detection and classification [40], medical image segmentation [41], and natural language processing [42]. Figure 3 shows the RNN structure. In order to avoid the problems of gradient vanishing or exploding, a long and short-term memory (LSTM) neural network, which involves creating a memory cell [43], is employed. Figure 4 shows the LSTM structure which illustrates the flow of data at time step *t*.

The cell state at time step *t* is given by

(2) $$c_{t}=f_{t}⊙c_{t-1}+i_{t}⊙g_{t}$$

where ⊙ stands for the Hadamard product (element-wise multiplication of vectors). The output (hidden) state at time step *t* is given by

(3) $$h_{t}=o_{t}⊙\tan h\left(c_{t}\right)$$

Here are the calculation procedures of the LSTM cell at time step t.

(4) $$i_{t}=\sigma \left(w_{i}x_{t}+R_{i}h_{t-i}+b_{i}\right)$$

(5) $$f_{t}=\sigma (w_{f}x_{t}+R_{f}h_{t-i}+b_{f}$$

(6) $$g_{t}=tanh\left(w_{g}x_{t}+R_{g}h_{t-1}+b_{g}\right)$$

(7) $$o_{t}=\sigma \left(w_{o}x_{t}+R_{o}h_{t-i}+b_{o}\right)\;$$

where $\sigma \left(.\right)$ stands for the sigmoid function given by $\sigma \left(z\right)={\left(1+e^{-z}\right)}^{-1}$, and x is the input of the time-series data.

In an LSTM layer, input weights w, recurrent weights R, and bias b need to be determined by learning. The matrices w, R, and b are concatenations of the input weights, the recurrent weights, and the bias of each component, respectively. The matrices are described as follows:

$$w=\left[\begin{array}{c} w_{i}\\ w_{f}\\ w_{g}\\ w_{o} \end{array}\right]\;\;R=\left[\begin{array}{c} R_{i}\\ R_{f}\\ R_{g}\\ R_{o} \end{array}\right]$$

and

$$b=\left[\begin{array}{c} b_{i}\\ b_{f}\\ b_{g}\\ b_{o} \end{array}\right]$$

where i, f, g, and o mark the input gate, forget gate, layer input, and output gate, respectively.

## 4. Fault Diagnosis of MMC-HVDC Systems with LSTM

### 4.1. Design of LSTM

The data used in this study are collected from a two-terminal simulation model of the MMC-HVDC transmission power system described in Section 2. Seven MMCs conditions include one normal condition and six IGBT open-circuit fault conditions in the lower and the upper arms of the MMC. A total of 100 examples of each condition, nine current signals of each example, and 5001 time samples of each current signal were recorded. Every current signal represents a time-series sample, so the fault information of MMC-HVDC systems is suitable for LSTM neural network.

The key parameters such as the number of layers, hidden layer size, batches, epochs, time steps, and learning rate, are very important to the performance of LSTM. In order to increase the model generalization ability and reduce the network calculation, we tested the different values of parameters. To minimize the error of the training, the backpropagation is used to update weights and bias. We selected the cross-entropy as the cost function to illustrate the error between the estimated value and the true value.

(8) $$E\left(\theta \right)=-\overset{N}{\underset{i=1}{\sum }}\overset{k}{\underset{j=1}{\sum }}t_{ij}ln(y_{j}\left(x_{i},\theta \right))$$

where $t_{ij}$ is the sign that the *i*-th example belongs to the *j*-th class, $y_{j}\left(x_{i},\theta \right)$ denotes the output for the *i*-th example.

*Adam* as a stochastic optimization method [44] is used to train LSTM and to determine network parameters, weights, and bias because *Adam* can adaptively adjust the learning rate by using the mean and variance of the gradient and has been successful in the learning rate optimization. *Adam* [44] uses an element-wise moving average of both the parameter gradients and their squared values to update the network parameters.

(9) $$\theta_{l+1}=\theta_{l}-\frac{\alpha m_{l}}{\sqrt{v_{l}}+\epsilon }$$

(10) $$m_{l}=\beta_{1}m_{l-1}+\left(1-\beta_{1}\right)𝛻E\left(\theta_{l}\right)$$

(11) $$v_{l}=\beta_{2}v_{l-1}+\left(1-\beta_{2}\right){\left[𝛻E\left(\theta_{l}\right)\right]}^{2}$$

where l denotes the iteration number, θ is the parameter vector, α is the learning rate, $\beta_{1}$ is the decay rate of gradient moving average, $\beta_{2}$ is the decay rate of squared gradient moving average, $𝛻E\left(\theta \right)$ is the gradient of the loss function, m is the first-moment estimate of the gradient, v is the second-moment estimate of the gradient, and ϵ is a small constant added to avoid division by zero. Here, we set α at 0.001, $\beta_{1}$ at 0.9, $\beta_{2}$ at 0.999, and $\epsilon =10^{-8}$.

### 4.2. Results and Analysis

In this section, the parameters of LSTM are selected, and the performance of the proposed method is illustrated and discussed.

#### 4.2.1. Parameters Selection of LSTM

To design an LSTM structure with higher classification accuracy, several parameters such as hidden layer size, mini-batch size, the maximum number of epochs, and learning rate, need to be discussed and determined. In this paper, the parameters quantification of hidden layer size, batches, and epochs have been explored to select better values based on a comparative evaluation of the performances. The learning rate is set to 0.001.

**Figure 5:**
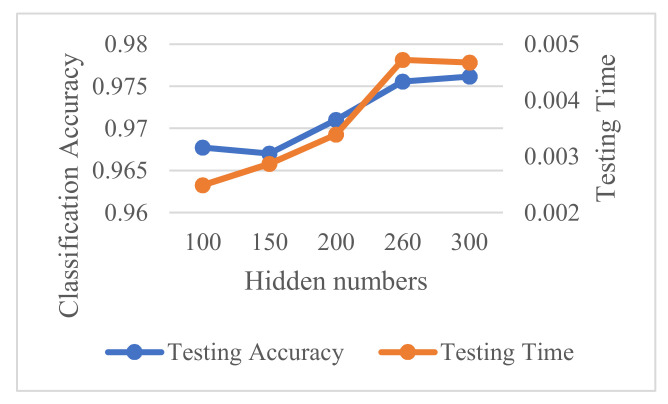
Accuracy and computation time at different hidden layer sizes.

The accuracy and computation time at different hidden layer sizes are depicted in Figure 5, when the maximum number of epochs set to 50 and the mini-batch size is set to 7. It can be shown that as the hidden layer size raises from 100 to 300, the computation time has a distinct peak with the hidden layer size set to 260 while the accuracy curve always rises as the layer size get bigger. In theory, the abscissa of the focus of the two lines should be the most optimal Hidden numbers. However, in the case of a small difference in time consumption, we are more concerned about the classification accuracy. Therefore, we select the hidden layer size as 300 by considering accuracy and computation time.

**Figure 6:**
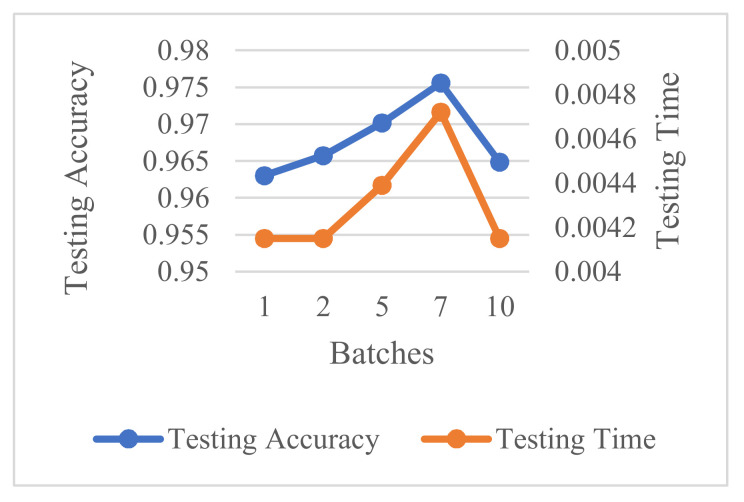
Accuracy and computation time at different mini-batch sizes.

The accuracy and computation time at different mini-batch sizes are depicted in Figure 6, when the maximum number of epochs set to 50 and the hidden layer size is set to 300. It can be shown that as the mini-batch size increases from 1 to 7, the accuracy curve and computation time have distinct rise, and then they tend to go down. We select the mini-batch size as 7.

**Figure 7:**
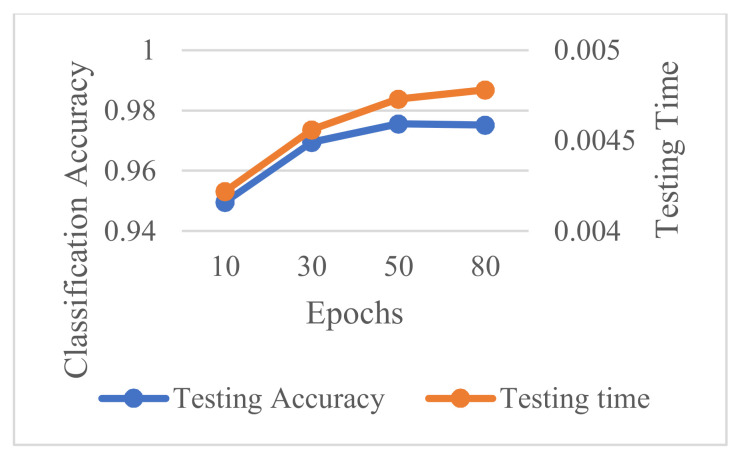
Accuracy and computation time at different maximum numbers of epochs.

The accuracy and computation time at different maximum numbers of epochs are depicted in Figure 7, when the hidden layer size is set to 300 and the mini-batch size is set to 7. It can be shown that as the maximum number of epochs raises from 10 to 80, the accuracy curve has a distinct peak with the maximum number of epochs set to 50 while the computation time curve always rises as the layer size get bigger. By considering comprehensively from both facets of accuracy and computation time, the maximum number of epochs is selected as 50.

#### 4.2.2. Detection and Classification of MMC-HVDC System with LSTM

According to the above studies, we set the parameters of LSTM hidden layer size at 300, the mini-batch size at 7, the maximum number of epochs at 50, and the learning rate at 0.001.

We conducted experiments from the testing data proportion of 0.1 to 0.9. For each testing data proportion, we ran it 20 times. The results following are the average of 20 runs. Testing data proportion is the ratio of the test samples number to the total number. The detection accuracy of the LSTM is described in Table 4. In terms of fault detection, the network output is divided into two types: normal and abnormal. We can see from Table 4 that the detection accuracy of the LSTM is 100% at each testing proportion.

**Figure 8:**
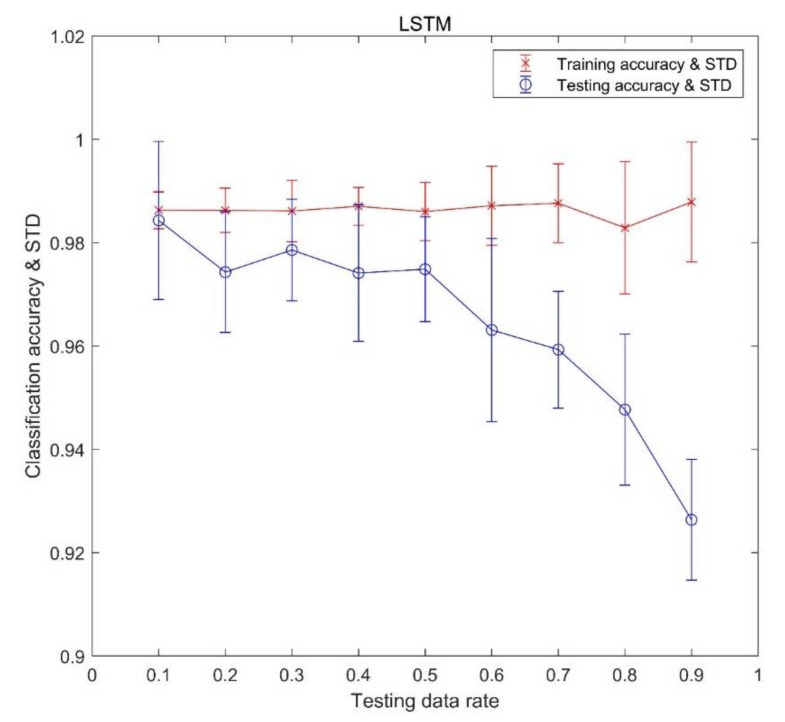
The classification results of training and testing data.

The results for training data and testing data are shown in Figure 8. STD in Figure 8 means the standard deviation, which is a measure that is used to quantify the amount of variation or dispersion of data values. It is observed that, with the rise of testing data proportion, classification accuracy for training data is steady (except a little dip at the testing data proportion of 0.8) and classification accuracy for testing data declines. The maximum mean accuracy of the testing dataset is 98.4% at 0.1 testing data proportion and the minimum average accuracy is 92.6% at 0.9 testing data proportion. The standard deviation of the classification accuracy for the training dataset increases with the increasing testing data proportion. However, for the testing dataset, the standard deviation of the classification accuracy at the ends of the testing data proportion is greater than around the middle of the testing data proportion. Moreover, the standard deviation of the classification accuracy for the training data set is less than that for the testing data set at all data proportions.

Table 5 is a confusion matrix of the classification results for each condition at testing data proportions of 0.2, 0.5, and 0.8. From Table 5, it is observed that the recognition of the normal condition is 100% at testing data proportions of 0.2, 0.5, and 0.8. At testing data proportion of 0.2, 2.75% of testing examples of B-phase lower SMs are misclassified as A-phase lower SMs and 1.5% of testing examples of B-phase lower SMs are misclassified as C-phase lower SMs. At testing data proportion of 0.5, 1.8% of testing examples of B-phase lower SMs are misclassified as A-phase lower SMs and 0.9% of testing examples of B-phase lower SMs are misclassified as C-phase lower SMs. Furthermore, at testing data proportion of 0.8, our method misclassified 1.69% of testing examples of B-phase lower SMs as A-phase lower SMs and 4.62% of testing examples of B-phase lower SMs as c C-phase lower SMs.

## 5. Comparison

To validate the effectiveness of the proposed method, several deep learning methods have been used for comparison. A Bidirectional LSTM (BiLSTM) is a sequence processing model that consists of two LSTMs: one access past information in a forward direction, and the other access future information in a reverse direction. The use of BiLSTM may not make sense for all sequence prediction problems but can offer some benefit in terms of better results to those domains where it is appropriate [45]. Therefore, we compare LSTM with BiLSTM on the detection accuracy, classification accuracy, training time spent, and the testing time spent with the testing data proportion from 0.1 to 0.9. We also compare it with CNN and AE-DNN. The implementation details of CNN and AE-DNN have been described in [33].

### 5.1. Comparison with BiLSTM

In order to compare, we set the parameters of BiLSTM the same as the parameters of the LSTM. The results are the arithmatic average of 20 runs, which include the detection accuracy, classification accuracy, training time spent, and the testing time spent. The comparisons are detailed in Table 6.

From Table 6, we can see that both LSTM and BiLSTM have the detection accuracy of 100%. The classification accuracy of BiLSTM is similar to LSTM, but BiLSTM required more training time and testing time.

### 5.2. Comparison with CNN and AE-DNN

**Figure 9:**
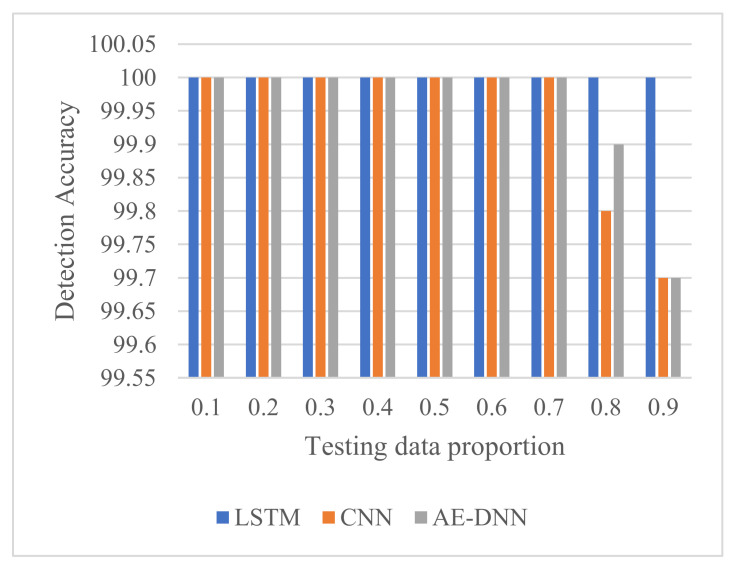
Comparison of detection accuracy.

Compared to CNN and AE-based DNN from Figure 9, it is observed that in terms of detection accuracy, the proposed method (LSTM) behaves outstandingly well at each testing data proportion. When the testing data proportion ranges from 0.1 to 0.7, these deep learning methods can detect faults perfectly.

**Figure 10:**
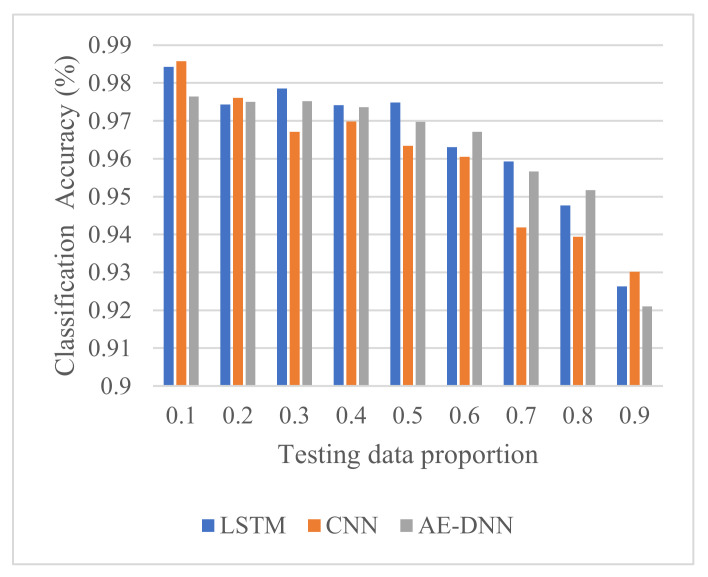
Comparison of classification accuracy.

Compared to CNN and AE-based DNN from Figure 10, it is observed that the proposed method (LSTM) offers higher classification accuracy at the testing data proportion 0.3, 0.4, 0.5, and 0.7. When the testing data proportion is 0.1, 0.2, and 0.9, which are located at the ends, CNN has better classification accuracy than LSTM and AE-based DNN.

**Figure 11:**
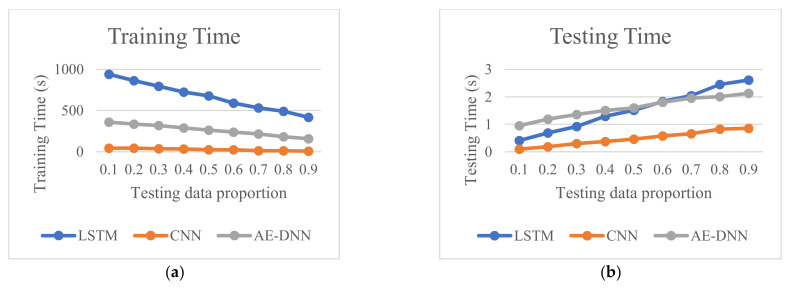
Comparison of computation time for the three methods. (**a**) Comparison of training time spent by the three methods, and (**b**) Comparison of testing time spent by the three methods.

Figure 11 shows the training time spent and testing time spent of the three methods. We can see that at each proportion, the LSTM method spends more training time than other methods and spends more testing time than CNN. We also can see that LSTM spends less testing time than AE-based DNN at the testing data proportions 0.1–0.6.

## 6. Conclusions

Fault diagnosis of MMC-HVDC has become one of the most important directions in research and practice. This paper presented an LSTM deep learning method for fault detection and classification to avoid the design of handcrafted features and classifiers. To validate its effectiveness, we compared it with BiLSTM and two other deep learning methods, CNN and AE-based DNN, using raw current sensor data of MMC-HVDC. The simulation results with data generated in PSCAD/EMTDC show that LSTM and BiLSTM have the best detection accuracy of 100%. CNN and AE-DNN can achieve high detection accuracy of more than 99.7%, while AE-based DNN is a little better than CNN. Additionally, these four methods achieve high classification accuracies. Compared with BiLSTM, LSTM has similar classification accuracy and requires less training time and less testing time. Compared with CNN and AE-DNN, LSTM provides better classification accuracy around the middle of the testing data proportions, though it needs more training time.

## Figures and Tables

**Table 1:** Output voltages of SM in normal and fault cases.

SM State	Normal	$T_{1}$ Fault	$T_{2}$ Fault
$S_{i}=1,i_{sm}>0$	$u_{c}$	$u_{c}$	$u_{c}$
$S_{i}=1,i_{sm}<0$	$u_{c}$	0	$u_{c}$
$S_{i}=0,i_{sm}>0$	0	0	$u_{c}$
$S_{i}=0,i_{sm}<0$	0	0	0

**Table 2:** Parameters of MMC [33].

Parameters	Value
number of SMs per arm	9
SM capacitor	1000 μF
arm inductance	50 mH
AC frequency	50 Hz

**Table 3:** MMC health conditions [33].

Faulty Bridge	Label Value
Normal	1
A-phase lower SMs	2
A-phase upper SMs	3
B-phase lower SMs	4
B-phase upper SMs	5
C-phase lower SMs	6
C-phase upper SMs	7

**Table 4:** Fault detection accuracy.

Testing Data Proportion	Detection Accuracy (%)
0.1	100
0.2	100
0.3	100
0.4	100
0.5	100
0.6	100
0.7	100
0.8	100
0.9	100

**Table 5:** Sample confusion matrix of the classification results.

**Testing Data Proportion = 0.2**							
	**Normal**	**A-Phase Lower SMs**	**A-Phase Upper SMs**	**B-Phase Lower SMs**	**B-Phase Upper SMs**	**C-Phase Lower SMs**	**C-Phase Upper SMs**
Normal	100	0	0	0	0	0	0
A-phase lower SMs	0	96.25	0	1.25	0	2.5	0
A-phase upper SMs	0	0	98.75	0	1.25	0	0
B-phase lower SMs	0	2.75	0	95.75	0	1.5	0
B-phase upper SMs	0	0	0	0	98.75	0	1.25
C-phase lower SMs	0	1.25	0	2.25	0	96.5	0
C-phase upper SMs	0	0	0.25	0	3.75	0	96
**Testing Data Proportion = 0.5**							
	**Normal**	**A-Phase Lower SMs**	**A-Phase Upper SMs**	**B-Phase Lower SMs**	**B-Phase Upper SMs**	**C-Phase Lower SMs**	**C-Phase Upper SMs**
normal	100	0	0	0	0	0	0
A-phase lower SMs	0	96.2	0	2.7	0	1	0.1
A-phase upper SMs	0	0	98.5	0	1.1	0	0.4
B-phase lower SMs	0	1.8	0	97.3	0	0.9	0
B-phase upper SMs	0	0.2	0.2	0	97.4	0	2.2
C-phase lower SMs	0	0.2	0	1.6	0	98	0.2
C-phase upper SMs	0	1.9	0	0	3.1	0	95
**Testing Data Proportion = 0.8**							
	**Normal**	**A-Phase Lower SMs**	**A-Phase Upper SMs**	**B-Phase Lower SMs**	**B-Phase Upper SMs**	**C-Phase Lower SMs**	**C-Phase Upper SMs**
normal	100	0	0	0	0	0	0
A-phase lower SMs	0	95.88	0	0.69	0	3.12	0.31
A-phase upper SMs	0	0	93.12	0.44	3.25	1.13	2.06
B-phase lower SMs	0	1.69	0	93.69	0	4.62	0
B-phase upper SMs	0	0.25	1.56	0	94.81	0.88	2.50
C-phase lower SMs	0	2.06	0	2.44	0	94.88	0.62
C-phase upper SMs	0	3.88	1.81	0	2.81	0.50	91

**Table 6:** The results comparison of LSTM with BiLSTM.

Testing Data Proportion	Detection Accuracy		Classification Accuracy		Training Time Spent		Testing Time Spent	
	LSTM	BiLSTM	LSTM	BiLSTM	LSTM	BiLSTM	LSTM	BiLSTM
0.1	100	100	0.984	0.974	942.4	2169.1	0.41	0.97
0.2	100	100	0.974	0.974	863.8	1519.0	0.69	1.40
0.3	100	100	0.979	0.980	794.4	1469.8	0.92	1.89
0.4	100	100	0.974	0.970	724.5	1362.9	1.29	2.63
0.5	100	100	0.975	0.973	677.9	1249.0	1.51	3.10
0.6	100	100	0.963	0.967	590.9	1147.0	1.83	3.93
0.7	100	100	0.959	0.962	531.6	1068.1	2.05	4.56
0.8	100	100	0.948	0.951	490.6	951.4	2.45	5.26
0.9	100	100	0.926	0.924	417.5	863.5	2.62	5.88

## Data Availability

The data presented in this study may be available on request from the first author, Q Wang. The data are not publicly available due to privacy reason.
